# Early predictors of late childhood behavioural outcomes following very preterm birth

**DOI:** 10.1017/S0033291725001151

**Published:** 2025-07-07

**Authors:** Zeyuan Sun, Andrew J Lawrence, Laila Hadaya, Alexandria O’Reilly Mescall, Lu Zhang, Qiaoyue Ge, Emily Simonoff, Serena J Counsell, A David Edwards, Paola Dazzan, Chiara Nosarti

**Affiliations:** 1 Department of Child and Adolescent Psychiatry, Institute of Psychiatry, Psychology and Neuroscience, King’s College London, London, UK; 2 Research Department of Early Life Imaging, School of Biomedical Engineering and Imaging Sciences, King’s College London, London, UK; 3 Department of Psychological Medicine, Institute of Psychiatry, Psychology, and Neuroscience, King’s College London, London, UK; 4 National Institute for Health Research (NIHR) Mental Health Biomedical Research Centre at South London and Maudsley NHS Foundation Trust and King’s College London, London, UK; 5 Department of Maternal and Child Health, West China School of Public Health and West China Fourth Hospital, Sichuan University, Chengdu, China

**Keywords:** behaviour, child, cognition, longitudinal studies, premature birth

## Abstract

**Background:**

Children born very preterm (VPT; ≤32 weeks’ gestation) are at higher risk of developing behavioural problems, encompassing socio-emotional processing and attention, compared to term-born children. This study aimed to examine multi-dimensional predictors of late childhood behavioural and psychiatric outcomes in very preterm children, using longitudinal clinical, environmental, and cognitive measures.

**Methods:**

Participants were 153 VPT children previously enrolled in the Evaluation of Preterm Imaging study who underwent neuropsychological assessments at 18–24 months, 4–7 years and 8–11 years as part of the Brain Immunity and Psychopathology following very Preterm birth (BIPP) study. Predictors of late childhood behavioural and psychiatric outcomes were investigated, including clinical, environmental, cognitive, and behavioural measures in toddlerhood and early childhood. Parallel analysis and exploratory factor analysis were conducted to define outcome variables. A prediction model using elastic-net regularisation and repeated nested cross-validation was applied to evaluate the predictive strength of these variables.

**Results:**

Factor analysis revealed two key outcome factors in late childhood: externalising and internalising-socio-emotional problems. The strongest predictors of externalising problems were response inhibition, effortful control and internalising symptoms in early childhood (cross-validated *R*^2^=.256). The strongest predictors of internalising problems were autism traits and poor cognitive flexibility in early childhood (cross-validated *R*^2^=.123). Cross-validation demonstrated robust prediction models, with higher accuracy for externalising symptoms.

**Conclusions:**

Early childhood cognitive and behavioural outcomes predicted late childhood behavioural and psychiatric outcomes in very preterm children. These findings underscore the importance of early interventions targeting cognitive development and behavioural regulation to mitigate long-term psychiatric risks in very preterm children.

## Introduction

Individuals born very preterm (VPT; ≤32 weeks’ gestation) are at higher risk of developing behavioural problems, encompassing socio-emotional functioning and attention, compared to term-born children (Delobel-Ayoub et al., 2006; Johnson & Marlow, 2011; Samuelsson et al., 2017). If these problems persist over time and are not addressed, they could lead to clinically significant psychiatric outcomes (Johnson et al., 2019). Recent work has shown that children born very preterm are more likely to receive a psychiatric diagnosis compared to their term-born peers: a ten-fold risk for autism spectrum disorder (ASD), a five-fold risk for attention deficit hyperactivity disorder (ADHD), a two-fold risk for anxiety disorder (AD), as well as a 50% higher risk of receiving a diagnosis of mood disorder (Anderson et al., 2021). Both clinical and subthreshold mental health problems have significant emotional and financial costs for families, and implications for public services, such as health insurance, education, and wider social support systems (Behrman & Butler, 2007). Therefore, early identification of behavioural problems in very preterm children and the provision of targeted interventions to support their development is vital for their well-being over the life course.

A major challenge for early identification of psychopathology is that often overt psychiatric symptoms do not manifest until childhood or even later, when individuals enter complex social environments, which may be overwhelming in the context of suboptimal socio-emotional development (Wolke, Johnson, & Mendonça, 2019). These findings point to the need to find the most significant predictors of behavioural and psychiatric outcomes early in life, to prevent behavioural problems from escalating and becoming clinically significant.

Previous studies have attempted to identify predictors of behavioural and psychiatric outcomes associated with very preterm birth with varying degrees of success, including clinical dimensions (e.g., gestational age and birth weight), environmental factors (e.g., family income, maternal education) and cognitive variables (e.g., intelligence and executive function) (Delobel-Ayoub et al., 2009; Treyvaud et al., 2013). However, these prediction models are either predominantly constrained by sample size or are not used in clinical practice (Linsell et al., 2017; Yaari et al., 2019). Models lacking measurements at different developmental stages and adequate age adjustment may also be unreliable, given that child development is a complex and dynamic process wherein environmental factors may gradually supersede the impact of biological factors as children grow (Cicchetti, 2016; Maggi, Irwin, Siddiqi, & Hertzman, 2010).

This study aimed to explore the most significant longitudinal predictors of late childhood behavioural and psychiatric outcomes in very preterm children, thereby providing insights for early identification and intervention. We hypothesised that environmental (e.g., maternal anxiety), cognitive (e.g., executive function, intelligence) and behavioural outcomes (e.g., temperament, internalising and externalising symptoms, autism traits) in toddlers (18–24 months) and young children (4–7 years) would predict later behavioural and psychiatric outcomes during late childhood (8–11 years).

## Methods

**Figure 1.**
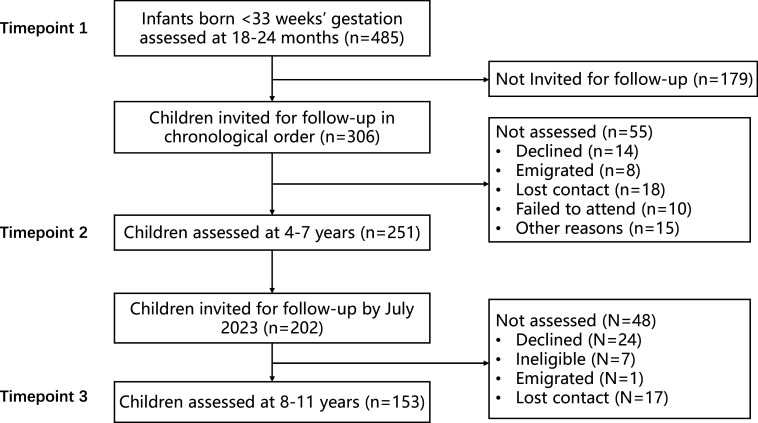
Recruitment flow chart.

Participants were very preterm children and their parents who took part in the Brain, Immunity and Psychopathology following very Preterm birth (BIPP) study. The BIPP study invited consenting participants who previously took part in the Evaluation of Preterm Imaging study (ePrime; EudraCT 2009-011602-42) (*n*=511) (Edwards et al., 2018) to complete a follow-up assessment between the ages of 8 and 11 years. 485 participants also completed neurodevelopmental assessments at 18–24 months (Edwards et al., 2018) and 251 participants at 4–7 years (Kanel et al., 2021; Vanes et al., 2021). Infants recruited into ePrime had the following inclusion criteria: birth before 33 weeks of gestation, maternal age above 16 years, and mothers not being hospital inpatients. Exclusion criteria were major congenital malformations, contraindications to magnetic resonance imaging, parents not being able to speak English, or being subject to child protection proceedings. Here we report data from 153 BIPP participants who completed the three assessments by July 2023 (Figure 1).

This study was conducted in line with the ethical standards of the 1964 Helsinki Declaration. The ePrime study was approved by the Hammersmith and Queen Charlotte’s Research Ethics Committee (09/H0707/98); BIPP by the South East Research Ethics Committee (19/LO/1940) and the Stanmore Research Ethics Committee (18/LO/0048).

### Behavioural outcome measures

Behavioural outcome measures were collected at three timepoints.

### 18–24 months assessment (T1)

Toddlers’ cognitive, motor and language development was indexed by five subscale scores of the Bayley Scales of Infant and Toddler Development–Third Edition (BSID-III) (Bayley, 2006) and two subscale scores of the Parent Report of Children’s Abilities-Revised (PARCA-R) (Johnson et al., 2004). Autism traits were evaluated with the Modified Checklist for Autism in Toddlers (M-CHAT) (Robins, Fein, & Barton, 1999), using the total number of failed items. Maternal anxiety was indexed by the State-Trait Anxiety Inventory (STAI) trait score (Spielberger, 1970).

### 4–7 years assessment (T2)

Children’s temperament was indexed by three subscale scores of the Children’s Behaviour Questionnaire – Very Short Form (CBQ-VSF) (Putnam, Gartstein, & Rothbart, 2006). Children’s mental health was indexed by the internalising and externalising problem scores of the Strengths and Difficulties Questionnaires (SDQ) (Goodman, 1997). Internalising difficulties are derived from the emotional and peer relationship problems subscales, while externalising difficulties are derived from the conduct and hyperactivity/inattention problems subscales (Goodman, Lamping, & Ploubidis, 2010). Autism traits were measured with the Social Responsiveness Scale Second Edition (SRS-2) (Constantino et al., 2003).

Cognitive variables were Intelligence Quotient (IQ), indexed by 12 item scores of the Wechsler Preschool and Primary Scale of Intelligence-fourth edition (WPPSI-IV) (Wechsler, 2012), everyday executive functions, indexed by three subscale scores of the Behaviour Rating Inventory of Executive Function, Preschool Version (BRIEF-P) (Gioia, Isquith, Guy, & Kenworthy, 2000), emotion regulation, indexed by the Emotion Regulation Checklist (ERC) (Shields & Cicchetti, 1997), attentional control, indexed by the Attention Network Task (ANT) (Rueda et al., 2004), cognitive flexibility, indexed by the Dimensional Change Card Sorting (DCCS) (Zelazo, 2006), working memory, indexed by Digit Span (using the Wechsler Intelligence Scale for Children [WISC] age-adapted normative scores), sustained attention, indexed by the Track-it! task (Fisher et al., 2013) and empathy, indexed by Empathy Questionnaire (Rieffe, Ketelaar, & Wiefferink, 2010). The cognitively stimulating home environment was indexed by our adapted version of the Cognitively Stimulating Parenting Scale (CSPS) (Vanes et al., 2021).

### 8–11 years assessment (T3)

Child’s temperament was indexed by three subscale scores of the Temperament in Middle Childhood Questionnaire, (TMCQ) (Simonds & Rothbart, 2004). Children’s mental health was indexed by SDQ internalising and externalising scores. Autism-related symptoms were indexed by the SRS-2. Anxiety symptoms were indexed by the Spence Children’s Anxiety Scale (SCAS) (Spence, 1998).

### Socio-demographic and clinical measures

The following socio-demographic and clinical measures were collected. Sex, ethnicity (defined by self-report based on classifications by the UK Office for National Statistics) (ONS, 2019), age at assessment, maternal education (categorized as “low” if she left full-time education before the age of 19, and “high” if she left full-time education after the age of 19) (Kleine et al., 2020) and relative social deprivation, indexed by the 2019 Index of Multiple Deprivation (IMD) (Ministry of Housing, 2019), calculated based on maternal postcode at recruitment. Gestational age (in weeks) and birth weight (in grams) were also collected.

### Statistical analysis

Statistical analysis was performed using R-4.3.1 (R Project for Statistical Computing). Descriptive results for categorical variables were presented as n (%) and continuous variables as means (standard deviations). Parallel analysis (R package *psych*) (Horn, 1965) and exploratory factors analysis (R package *lavaan*) (Rosseel, 2012) were conducted to reduce the dimensionality of the outcomes. The code used can be accessed here: (https://github.com/RobinZSun/dCVnet_BIPP). Further details on variable characteristics can also be found in Supplementary Table 1. The study variables exhibited missingness ranging from 0% to 23.53% across predictors, while the missingness for outcome variables ranged from 6.54% to 45.75% (complete distributions provided in Supplementary Table 1). Formal testing using Jamshidian and Jalal’s (2010) multivariate Missing Completely At Random procedure rejected the missing completely at random assumption (median *χ*^2^=25.29, *p*=0.01), supporting our missing-at-random working assumption. All missing data were jointly imputed using missForest’s random forest approach (Stekhoven & Buhlmann, 2012). To confirm the representativeness of the study sample, demographic and clinical factors were compared between participants included in the current analysis (*N* = 153) and all who completed the T2 assessments (*N* = 251) (See Supplementary Table 2). There were no significant differences in terms of sex distribution (*χ* = 0.01, *p* = 0.93), IMD (*t* = −0.71, *p* = 0.48) and GA (*t* = −0.89, *p* = 0.37) between the two samples.

### Prediction modelling

The seven separate childhood outcome variables (three TMCQ subscale scores, SCAS total score, two SDQ broad categories and SRS-2 total score) were included in an exploratory factors analysis. The derived factors were used as outcomes for the prediction models, and separate prediction models were built for each outcome factor. All cognitive and behavioural measures collected at T1 and T2 were included as predictors. We excluded any predictors with pre-imputation missing values greater than 20% (two variables). To ensure our models prediction performance did not reflect trivial effects of assessment timing and demographic factors, both predictors and outcome factors were adjusted for age at assessment, while predictors were further adjusted for sex, ethnicity, mother’s education, IMD and gestational age. To retain the most significant longitudinal predictors of outcomes, we used an approach that accommodates a wide range of predictors and their combinations, while eliminating redundant predictors from the model. This approach is called double cross-validation for the elastic-net (dCVnet); R package dCVnet (https://www.github.com/AndrewLawrence/dCVnet) developed by coauthor AL (Lawrence et al., 2022). The dCVnet software fits regression models with elastic net-regularization and provides internal validity of the prediction performance using double (also known as “nested”) cross-validation (repeated k-fold type). This combines an inner cross-validation, which tunes elastic net hyperparameters, with an outer cross-validation of model performance measures. This ensures the validity of the cross-validation measures (Cawley & Talbot, 2010), while the regularisation performs data-driven variable selection, reduces overfitting, and allows models with correlated predictors (Zou & Hastie, 2005). The model utilises simple linear functions of predictors and is more directly interpretable than complex machine-learning techniques (Christodoulou et al., 2019). For each outcome factor, the prediction models used a gaussian family elastic-net with alpha = 0.2 and a data-derived sequence of 100 lambdas on a log scale. Internal cross-validation was 30×10-fold, while external cross-validation was 100×10-fold. The cross-validated prediction performance was evaluated by three indicators: the Root-Mean-Square Error (RMSE), the Mean-Absolute Error (MAE) and R square (*r*^2^).

## Results

### Sample characteristics

Sample characteristics are shown in Table 1.

**Table 1.** Clinical, socio-demographic and parental characteristics of the sample (*n* = 153)

Variable	Description
Sex	*n* (%)
Male	82 (53.59)
Female	71 (42.41)
Ethnicity	
Caucasian	96 (62.75)
Asian	22 (14.38)
Black	16 (10.45)
Other	19 (12.42)
Maternal education^a^	
Low	32 (20.92)
High	121 (79.08)
	Median [Range]
Gestational age (week)	29.71 [23.86, 32.86]
Birth weight (g)	1250 [600, 2160]
IMD score	15.90 [1.85, 61.25]
Age at assessment 1 (month)	20.00 [19.00, 32.86]
Age at assessment 2 (year)	4.64 [4.18, 6.95]
Age at assessment 3 (year)	9.16 [7.00, 12.42]

a “Low” indicates exiting full-time education by 19 years, “High” indicates exiting full-time education later than 19 years or still in full-time education.

### Factor analysis of behavioural outcomes in late childhood

**Figure 2.**
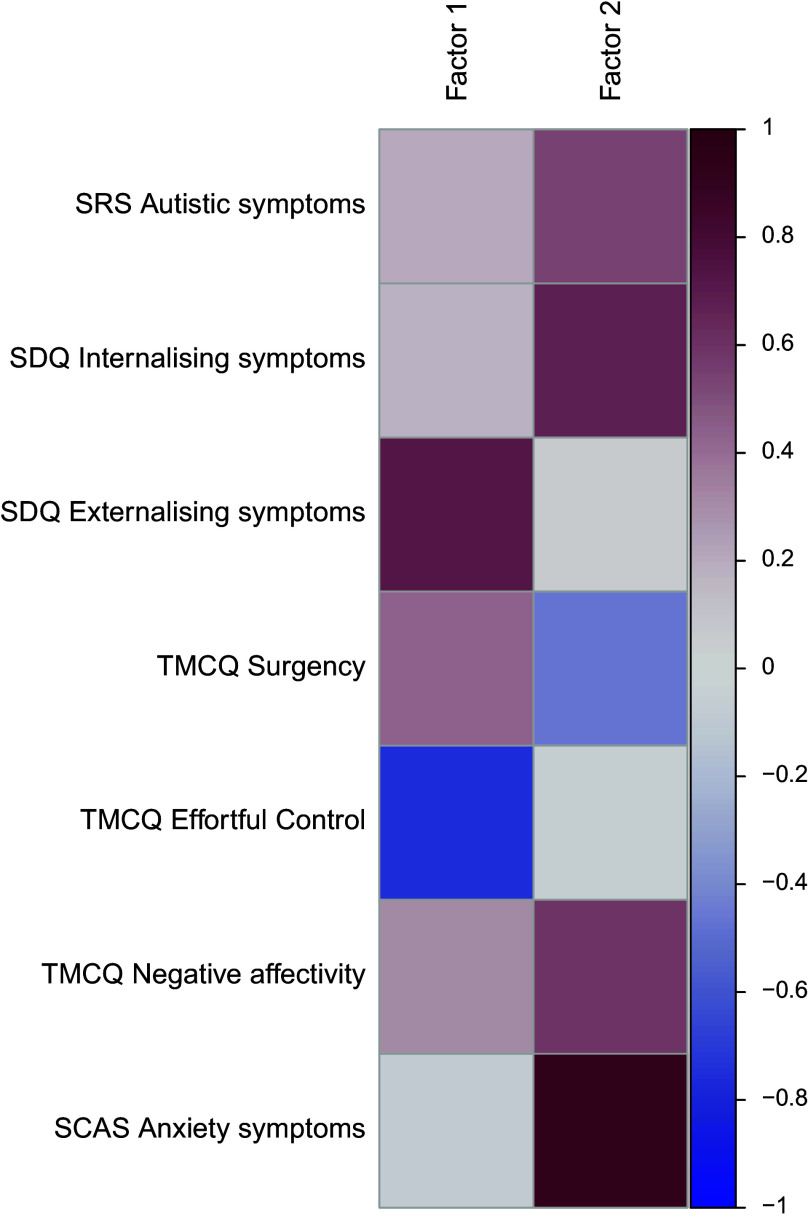
Heatmap of loadings of each variable on Factor 1 (externalising problems) and Factor 2 (internalising-socio-emotional problems). Positive loadings (>0) are indicated by red and negative loadings (<0) by blue.

Parallel analysis and exploratory factor analysis on outcome variable identified two factors, jointly explaining a cumulative 54.9% of the total variance. Individual loadings of outcome variables for these two factors are depicted in Figure 2.

Factor 1 was driven by positive loadings for measures capturing externalising symptoms (SDQ externalising symptoms), Surgency and Negative Affectivity (TMCQ), as well as negative loadings for measures capturing impulse control (TMCQ Effortful control). We labelled this factor as “Externalising”.

Factor 2 was driven by positive loadings for measures capturing internalising symptoms (SDQ internalising symptoms), autism traits (SRS-2), anxiety symptoms (SCAS) and Negative Affectivity (TMCQ), as well as negative loadings for Surgency (TMCQ). We labelled this factor as “Internalising-socio-emotional”.

### Prediction of behavioural and mental health outcomes in late childhood

Cross-validation revealed robust, better than chance, prediction of both “externalising” and “internalising-social-emotional” outcomes (Table 2). Model coefficients (Table 3) suggest that increased externalising problems in late childhood were associated with increased maternal anxiety in toddlerhood (maternal STAI), poorer verbal concept formation and reasoning (WPPSI similarities), working memory (WPPSI picture memory and digit span), emotion regulation, increased autistic traits (SRS-2) and more behavioural problems in early childhood (CBQ surgency and SDQ internalising problems), with the most important predictors being poorer inhibitory self-control, emergent metacognition, more externalising problems and reduced effortful control. They also show that internalising-socio-emotional problems in late childhood were associated with more autistic traits in toddlerhood (M-Chat), poorer general cognitive ability (WPPSI information) and more internalising problems, with the most important predictors being poorer cognitive flexibility, increased autistic traits (SRS-2) and lower surgency (Table 3).

**Table 2.** Model performance measures of cross-validations (*k* = 10, lambda = 100)

Outcome	Model measure	Mean (Standard error)	
		Reference (intercept-only)	Observed
Externalising problems	RMSE	0.997 (–)	0.751 (0.005)
	MAE	0.996 (0.085)	0.588 (0.004)
	*r*^2^	–	0.256 (0.012)
Internalising-social-emotional problems	RMSE	0.998 (–)	0.882 (0.007)
	MAE	0.998 (0.211)	0.592 (0.005)
	*r*^2^	–	0.123 (0.015)

*Note:* RMSE, Root-Mean-Square Error; MAE, Mean-Absolute Error; *r*^2^, R square.

**Table 3.** Prospective predictors of behavioural and mental health outcomes in late childhood

Time	Predictor	Externalising problems factor (β)	Internalising-socio-emotional problems factor (β)
T1	Maternal STAI	0.02	–
	M-Chat total	–	0.03
T2	WPPSI information	–	−0.02
	WPPSI similarities	−0.02	–
	WPPSI picture memory	−0.02	–
	BRIEF inhibitory self-control	**0.11**	–
	BRIEF flexibility	–	**0.10**
	BRIEF emergent metacognition	**0.07**	–
	Digit span	−0.04	–
	Emotional regulation checklist	−0.05	–
	SRS–2	0.05	**0.15**
	CBQ surgency	0.02	**−0.06**
	CBQ effortful control	**−0.12**	–
	SDQ internalising	0.04	0.02
	SDQ externalising	**0.07**	–

*Note*: hyphen (“-”) indicates variables assigned a coefficient of exactly zero and so not selected in the model. The largest magnitude coefficients (greater than |0.06|) are highlighted in bold. In addition, the following predictors were included, but not selected by either model: BSID-III cognitive subscale, BSID-III expressive language subscale, BSID-III receptive language subscale, BSID-III fine motor subscale, BSID-III gross motor subscale, PARCA_R cognition subscale, PARCA_R language subscale, WPPSI block design, WPPSI matrix reasoning, WPPSI bug search, WPPSI picture concepts, WPPSI cancellation, WPPSI zoo location, WPPSI object assembly, WPPSI receptive vocabulary, WPPSI picture naming, CBQ negative affectivity, Track it! memory, Track it! attention, Card sort total, ANT correct percentage, Emque total, CSPS total. For variable characteristics see Supplementary Table 1.

## Discussion

In this study, we explored the most important longitudinal predictors of late childhood behavioural and psychiatric outcomes in very preterm children. We identified two behavioural and psychiatric phenotypes in late childhood, the first characterised by externalising problems and the second by internalising-social-emotional problems. Analyses investigated previously collected variables (cognitive, behavioural, parental measures in toddlerhood and early childhood) as predictors of behavioural and psychiatric outcomes in later childhood. Our findings indicated that greater maternal anxiety, poorer executive function, verbal concept formation and reasoning, working memory, emotion regulation, effortful control, more autistic traits and behavioural problems during early childhood were associated with externalising problems in later childhood. They also showed that more autistic traits in toddlerhood and early childhood, poorer general cognitive ability and flexibility, lower surgency and more internalising problems, were associated with internalising-social-emotional problems in later childhood.

Our findings are in line with previous studies investigating associations between mental health outcomes and cognitive, behavioural and parental measures separately (Bayer et al., 2011; Pettersson, Lahey, Larsson, & Lichtenstein, 2018). Behavioural and mental health outcomes were summarised into “externalising” and “internalising-social-emotional” problems, which incorporated multi-dimensional mental health features consistent with common psychiatric outcomes during late childhood (Johnson & Marlow, 2011; Treyvaud et al., 2013). The “externalising” factor used in our analyses comprised externalising symptoms (conduct problems and hyperactivity), surgency, referring to low shyness, high-intensity pleasure and impulsiveness (Oldehinkel et al., 2004), negative affectivity, referring to the tendency to attribute negative feelings to contexts, and overexpression of negative affect (Putnam et al., 2001) and lack of effortful control, referring to individuals’ ability to direct their attention and regulate their emotions and behaviours (Damon, Lerner, & Eisenberg, 2006). In contrast, the “internalising-social-emotional” factor comprised autistic traits, internalising symptoms (emotional and peer problems), negative affectivity, anxiety symptoms and low surgency. Notably, negative affectivity loaded on both factors, suggesting that susceptibility to negative emotions may be a common underlying feature (Engle & McElwain, 2011). On the other hand, surgency loaded on both factors yet in opposite directions, which is in line with previous findings (Secinti & Gürbüz, 2021). The performance of the prediction model for externalising problems was slightly better than that for internalising-social-emotional problems. This could be because externalising symptoms tend to have a higher intensity than the internalising ones in late childhood (Papachristou & Flouri, 2020), and were therefore easier to observe and measure in this study.

Our findings showed that the most important predictor of late childhood internalising-socio-emotional problems among very preterm children was a higher level of autism traits in early childhood, which is consistent with prior research (Mandy et al., 2022). The association between early autism traits and later internalising problems has been the focus of previous studies. From a biological perspective, shared genetic influences between autistic-like traits and emotional and behavioural problems have been reported (Hollo, Wehby, & Oliver, 2014; Ronald, Edelson, Asherson, & Saudino, 2010), indicating potential shared biological pathways. However, environmental factors have also been put forward to understand the association. For example, social opportunities provided by parents may influence children’s social and communication skills. Children with poorer social communication skills demonstrated more autistic symptoms compared to those with better social communication abilities (Craig et al., 2021), which could lead to a higher level of emotional problems (Salomone et al., 2019). Another study also suggested that earlier communication and social interaction difficulties may mediate the associations between autism traits and subsequent internalising problems (Hallett, Ronald, Rijsdijk, & Happe, 2010; Stice & Lavner, 2019). Communicative impairments can confuse a child’s understanding of others, limit self-expression, and reduce coping strategies for negative emotions, such as anxiety and sadness (Donoso et al., 2023).

We found associations between low effortful control and later externalising problems; and low surgency and later internalising-socio-emotional problems. These findings indicate consistency in temperamental traits from early to late childhood, as we used the same scales for both predictor (CBQ) and outcome (TMCQ) at different ages (Putnam, Gartstein, & Rothbart, 2006). Research findings suggest that children’s temperaments are relatively stable across developmental stages (Komsi et al., 2006), mirroring our findings – where the effect of three temperamental types in early childhood (captured by the CBQ) aligns with those observed in later childhood (captured by the TMCQ). Our findings support previous studies linking temperament and behavioural problems. For instance, effortful control plays an important role in shaping psychological and social adjustment (Meehan, De Panfilis, Cain, & Clarkin, 2013). High effortful control has been linked to a reduced risk of externalising problems (Oldehinkel et al., 2007) and low effortful control to increased behavioural problems (Atherton, Zheng, Bleidorn, & Robins, 2019). Low surgency has been associated with a greater likelihood of exhibiting behavioural wariness, with emotion regulation playing a moderating role (Dollar & Stifter, 2012). It has also been suggested that there may be transactional effects between maternal anxiety and child effortful control (Behrendt et al., 2020): maternal anxiety could lead to low control in children and vice versa, and this interaction further contributes to subsequent behavioural problems in offspring.

In terms of executive function, we found that poorer inhibitory self-control in early childhood was associated with more externalising problems in later childhood, while poorer cognitive flexibility in early childhood was linked to internalising social-emotional problems in later childhood. Genetic studies have shown that the relationship between response inhibition and externalising problems could be explained by both genetic and shared environmental influences (Rhee et al., 2018). Another study suggested that the impact of poor response inhibition on behavioural problems was related to its association with disruptive social competence in primary school children (Wang & Liu, 2021). At a biological level, research indicates that enhanced parasympathetic activity may serve as a common pathway underlying both poor self-control and externalising problems in children (Kahle, Utendale, Widaman, & Hastings, 2018). On the other hand, children showing good cognitive flexibility skills may be less likely to develop internalising behaviour problems (Patwardhan, Nelson, McClelland, & Mason, 2021). Cognitive flexibility may enable children to shift between activities, allowing them to disengage from negative stimuli and aversive situations, and instead focus selectively on more positive thoughts (Garon, Bryson, & Smith, 2008). More broadly, associations between executive functions and behavioural problems are frequently reported; however, the directionality of these associations remains uncertain (Donati, Meaburn, & Dumontheil, 2021; Yang et al., 2022). Inconsistent findings may be due to variability in the measurements used to assess executive functions and behavioural problems, participant characteristics (e.g., age groups, clinical vs. general populations), and the specific behavioural problems studied (e.g., ADHD, ASD, externalising behaviour) (Yang et al., 2022). A recent study indicated that executive function is both a risk marker and a consequence of youth transdiagnostic psychopathology (Romer & Pizzagalli, 2021). There is evidence that general psychopathology and executive dysfunction may share neural circuits, specifically within the frontoparietal network, visual association cortex, and cerebello-thalamo-cerebro-cortical pathways (Alnaes et al., 2018; Karcher, Michelini, Kotov, & Barch, 2021; Moberget et al., 2019).

Noticeably, both internalising and externalising symptoms in early childhood were associated with externalising problems in later childhood, while only internalising symptoms in early childhood were associated with internalising-socio-emotional problems in later childhood. This may be due, at least in part, to the way our outcomes of interest were defined based on the results of the factor analysis. The externalising factor captures conduct problems, hyperactivity, low impulse control but also negative affectivity, which has been associated with both internalising and externalising disorders in children (Mikolajewski et al., 2013). On the other hand, the internalising-socio-emotional problems factor consists of a broad set of difficulties in social engagement and emotional regulations (e.g., autistic traits, emotional and peer problems, anxiety symptoms and susceptibility to negative emotions), hence it is plausible that early conduct problems and hyperactivity (i.e., SDQ externalising symptoms) may not contribute to the development of internalising-socio-emotional problems in later childhood. Our findings can also be explained by the stability of internalising and externalising symptoms, as externalising symptoms in childhood have been reported to be more transient and worsen over time, whereas internalising symptoms tend to show more consistency (Blok et al., 2022). Furthermore, internalising symptoms have been found to predict later internalising outcomes, whereas externalising symptoms have not been shown to predict externalising outcomes (Sallis et al., 2019).

We found that poorer verbal concept formation, reasoning, working memory and emotion regulation in early childhood were associated with more externalising problems in later childhood, while poorer general cognitive ability was prospectively associated with internalising-socio-emotional problems. Despite limited evidence, it has been suggested that reduced performance in reading, writing, and spelling may be associated with emotional and behavioural problems in children (Khanam & Nghiem, 2018). Among the various mediating factors, parental education and school performance appear to play a significant role in this association (Tamayo Martinez et al., 2021). The relationship between externalising problems and cognitive abilities, such as working memory (Augusti, Torheim, & Melinder, 2014; Huang-Pollock, Shapiro, Galloway-Long, & Weigard, 2017) and emotional regulation (Augusti, Torheim, & Melinder, 2014), has been widely documented. Working memory deficits may contribute to poor academic outcomes and result in children’s exposure to negative emotions arising from teachers’ feedback (Lahey et al., 2015), which in turn can lead to externalising problems (Tajik-Parvinchi et al., 2021).

We found that increased maternal anxiety in toddlerhood was associated with increased externalising problems in offspring, in line with previous findings (Spence et al., 2002). The precise mechanisms underlying the association between maternal anxiety and children’s outcomes are not well understood. One of the most accepted theories suggests that this influence occurs via the maternal hypothalamic-pituitary-adrenal axis, leading to higher cortisol levels, higher placental corticotropin-releasing hormone levels and reduced blood serum levels of placental growth factor (Hobel et al., 1999; Torry et al., 1998). These stress-related hormones could interfere with the development of the fetal brain and affect offspring socioemotional and behavioural functioning across the lifespan (Hostinar & Gunnar, 2013). In addition, though most prior studies cannot rule out genetic confounding as an explanation, some evidence suggests that maternal prenatal stress may lead to children’s behavioural problems by influencing the family environment in the postnatal period (Rice et al., 2010). We did not find an association between maternal anxiety during toddlerhood and internalising or socio-emotional problems in offspring, which contradicts much of the existing literature (Henrichs et al., 2019; Shih et al., 2023). This discrepancy may be partially due to the type of behavioural problems at this stage, manifesting more as externalising rather than internalising symptoms. Additionally, the timing of the assessment and the choice of measure (STAI traits) may confound the interpretation of the effects observed. Finally, the broad nature of the internalizing-socio-emotional problems factor in our study, encompassing a wide range of difficulties beyond internalising symptoms, may explain why maternal anxiety may not directly influence their development in late childhood.

Previous studies building prediction models for psychiatric outcomes during childhood among preterm children usually included only a limited number of predictors and outcomes (Anderson et al., 2021; Yaari et al., 2019). We applied a mixed approach of double-cross validation and elastic net regularisation to allow a greater number of correlated predictors without overfitting and without losing model interpretability. By incorporating the indicators identified from infancy through early childhood into routine paediatric developmental screenings, clinicians could enhance risk stratification. An updated framework that emphasises transdiagnostic risk factors, rather than symptom-based screening, may enable earlier detection through fewer, yet more precise, indicators. In high-risk populations (e.g., very preterm children), these predictors could also serve as potential indicators for evaluating early interventions.

Several limitations should be considered when interpreting the findings of our study. Firstly, the outcomes of the prediction model were based on the results of a factor analysis. Therefore, the interpretation of the model relies more on the clinical significance of the two outcome factors than on the standardised scales that comprised them. Secondly, our analysis was based on a comparatively small sample size with a rather large set of variables, which limits model performance and the generalisation of our findings. Thirdly, some variables were more affected by missing data than others (see Supplementary Table 1). Although the imputation method we used (MissForest) outperforms other methods for the imputation of missing values in mixed-type data (Stekhoven & Buhlmann, 2012), those predictors with fewer missing values may nevertheless be favoured by prediction models (Moons, Donders, Stijnen, & Harrell, 2006). A further limitation is the lack of granular information about family-level contextual factors (e.g., caregiver relationships, parenting practices, or household dynamics) (Fosco & Lydon-Staley, 2020; Hosokawa & Katsura, 2024), which were not fully captured by our measures. Finally, our study was based on longitudinal cognitive and behavioural measurements and did not include data on children’s brain development, which prevented the study from probing the biological basis of the outcome measures of interest. We consider these two models as baseline frameworks, which we hope future analyses incorporating neuroimaging data will further refine.

## Conclusion

We identified two phenotypes of late-childhood behavioural problems, derived from several common mental health outcomes in children born very preterm, and developed two separate prediction models accordingly. Our findings suggest that poorer inhibitory self-control and cognition, along with heightened externalising symptoms in early childhood, predict externalising problems in late childhood. Additionally, poor cognitive flexibility, increased internalising symptoms and autism traits during early childhood predict internalising social-emotional problems in late childhood. Future research should evaluate their clinical utility in guiding interventions designed to mitigate the long-term sequelae of very preterm birth, thereby improving developmental trajectories and overall well-being in very preterm children.

## Supporting information

Sun et al. supplementary material Sun et al. supplementary material

## Data Availability

The data that support the findings of this study are available on request from the corresponding author. The data are not publicly available due to ethical restrictions.

## Supplementary material

The supplementary material for this article can be found at http://doi.org/10.1017/S0033291725001151.
