# Supplementary material for: Early predictors of late childhood behavioural outcomes following very preterm birth
Source: Psychol Med. 2025 Jul 7;55:e189. doi: 10.1017/S0033291725001151 (PMC12270274; doi:10.1017/S0033291725001151)
Supplement: Sun et al. supplementary material [file S0033291725001151sup001.docx]

**Supplementary Table 1: Socio-demographic, clinical, cognitive and behavioural variables of study participants**

| **Variable name (Unit)** | | **Mean (SD)** | | **Range** | | **Missing proportion (%)** | |
| --- | --- | --- | --- | --- | --- | --- | --- |
| Sex | | **n** | | **Percentage** | | 0.00 | |
| Male | | 82 | | 53.59 | |  | |
| Female | | 71 | | 42.41 | |  | |
| Ethnicity | | **n** | | **Percentage** | | 7.19 | |
| Caucasion | | 86 | | 56.21 | |  | |
| Asian | | 21 | | 13.73 | |  | |
| Black | | 16 | | 10.46 | |  | |
| Other | | 19 | | 12.42 | |  | |
| Maternal education | | **n** | | **Percentage** | | 0.00 | |
| Low | | 32 | | 20.92 | |  | |
| High | | 121 | | 79.08 | |  | |
| IMD | | 18.57 (11.84) | | [1.85, 61.25] | | 0.00 | |
| Gestational age (weeks) | | 29.49 (2.30) | | [23.86, 32.86] | | 0.00 | |
| Age at assessment 1 (year) | | 1.67 (0.06) | | [1.58, 2.08] | | 0.00 | |
| Age at assessment 2 (year) | | 4.97 (0.68) | | [4.18, 6.95] | | 0.00 | |
| Age at assessment 3 (year) | | 9.35 (1.10) | | [7.00, 12.42] | | 0.00 | |
| **T1: 22 months assessment** | |  | |  | |  | |
| Maternal STAI | | 36.29 (10.36) | | [20, 59] | | 16.99 | |
| BSID-III cognitive subscale | | 54.59 (5.40) | | [38, 66] | | 0.00 | |
| BSID-III expressive language subscale | | 21.56 (4.58) | | [10, 32] | | 0.00 | |
| BSID-III receptive language subscale | | 22.89 (5.49) | | [10, 35] | | 0.00 | |
| BSID-III fine motor subscale | | 36.38 (2.74) | | [27, 43] | | 0.00 | |
| BSID-III gross motor subscale | | 49.9 (5.05) | | [22, 57] | | 0.00 | |
| PARCA_R cognition subscale | | 22.32 (5.22) | | [8, 31] | | 1.31 | |
| PARCA_R language subscale | | 26.46 (20.13) | | [0, 94] | | 1.31 | |
| M-Chat Total | | 1.78 (1.96) | | [0, 12] | | 0.00 | |
| **T2: 4-7 years assessment** | |  | |  | |  | |
| WPPSI Block Design | | 19.97 (4.17) | | [6, 32] | | 1.31 | |
| WPPSI Information | | 19.71 (3.23) | | [6, 26] | | 1.96 | |
| WPPSI Matrix Reasoning | | 11.43 (4.23) | | [3, 22] | | 1.96 | |
| WPPSI Bug Search | | 26.61 (9.76) | | [0, 56] | | 1.96 | |
| WPPSI Picture Memory | | 15.92 (5.21) | | [2, 30] | | 1.96 | |
| WPPSI Similarities | | 20.59 (8.17) | | [3, 38] | | 2.61 | |
| WPPSI Picture Concepts | | 11.58 (4.76) | | [0, 21] | | 3.27 | |
| WPPSI Cancellation | | 33.1 (12.69) | | [0, 66] | | 1.96 | |
| WPPSI Zoo Location | | 10.34 (2.35) | | [3, 16] | | 2.61 | |
| WPPSI Object Assembly | | 22.27 (8.29) | | [0, 35] | | 0.00 | |
| WPPSI Receptive Vocabulary | | 20.12 (4.81) | | [5, 31] | | 0.00 | |
| WPPSI Picture naming | | 16.82 (2.55) | | [6, 23] | | 0.65 | |
| BRIEF Self-Control Index | | 42.41 (10.46) | | [26, 76] | | 1.96 | |
| BRIEF Flexibility Index | | 30.27 (7.37) | | [20, 58] | | 1.96 | |
| BRIEF Emotional Regulation Index | | 42.91 (10.66) | | [27, 80] | | 1.96 | |
| SRS | | 39.53 (25.93) | | [1, 153] | | 5.88 | |
| CBQ Surgency | | 4.72 (0.84) | | [2.08, 6.42] | | 2.61 | |
| CBQ Negative Affectivity | | 4.11 (0.91) | | [1.58, 6] | | 2.61 | |
| CBQ Effortful Control | | 5.27 (0.79) | | [2.25, 6.92] | | 2.61 | |
| SDQ internalising | | 3.79 (2.77) | | [1, 13] | | 19.61 | |
| SDQ externalising | | 6.46 (3.88) | | [1, 16] | | 7.84 | |
| Digit span total recall | | 7.49 (3.21) | | [1, 15] | | 7.19 | |
| ERC total | | 33.74 (6.48) | | [9, 45] | | 5.23 | |
| Track it! memory | | 7.46 (3.01) | | [0, 10] | | 22.22 | |
| Track it! attention | | 7.17 (3.20) | | [0, 10] | | 23.53 | |
| DCCS total | | 41.95 (3.17) | | [32, 48] | | 5.88 | |
| ANT correct percentage | | 81.35 (17.32) | | [10.42, 98.96] | | 12.42 | |
| Emque total | | 0.87 (0.26) | | [0.25, 1.55] | | 2.61 | |
| CSPS total | | 17.56 (2.55) | | [9, 21] | | 3.27 | |
| **T3: 8-11 years assessment** | |  | |  | |  | |
| SDQ internalising | | 4.97 (3.45) | | [0, 15] | | 6.54 | |
| SDQ externalising | | 5.87 (3.31) | | [0, 17] | | 6.54 | |
| TMCQ Surgency | | 3.14 (0.52) | | [1.59, 4.29] | | 11.11 | |
| TMCQ Effortful Control | | 3.33 (0.46) | | [2.18, 4.36] | | 11.11 | |
| TMCQ Negative Affectivity | | 2.32 (0.62) | | [1.06, 4.13] | | 11.11 | |
| SRS | | 58.81 (34.02) | | [4, 238] | | 9.80 | |
| SCAS total | | 17.83 (13.00) | | [2, 81] | | 45.75 | |

**Supplementary Table 2. Comparing socio-demographic and clinical variables between the T2 and T3 samples**

| Variable | T2 sample (N=251) | T3 sample (N=153) | Test value | P value |
| --- | --- | --- | --- | --- |
| Gestational age (week), mean (SD) | 29.70 (2.29) | 29.49 (2.30) | -0.89 | 0.372 |
| IMD score, mean (SD) | 19.45 (12.26) | 18.57 (11.84) | -0.71 | 0.475 |
| Sex, n (%) |  |  |  |  |
| Male | 132 (52.59) | 82 (53.59) | 0.01 | 0.925 |
| Female | 119 (47.41) | 71 (42.41) |  |  |

*Table Caption: T2 sample = participants who completed the 4-7 assessments. T3 sample = participants who completed the 8-11 assessments by July 2023. T-test was conducted for gestational age and IMD score and Chi-square test for sex.*

**Supplementary Table 3. Loadings matrix of the outcome measure for the derived factors**

| **Outcome measures** | **Factor 1 (Externalising problems)** | **Factor 2 (Internalising-social-emotional problems)** |
| --- | --- | --- |
| SRS Autistic symptoms | 0 | 0.548 |
| SDQ Internalising symptoms | 0 | 0.677 |
| SDQ Externalising symptoms | 0.730 | 0 |
| TMCQ Surgency | 0.438 | -0.465 |
| TMCQ Effortful Control | -0.761 | 0 |
| TMCQ Negative affectivity | 0.317 | 0.594 |
| SCAS Anxiety symptoms | 0 | 0.932 |
